# Fear of Falling Does Not Influence Dual-Task Gait Costs in People with Parkinson’s Disease: A Cross-Sectional Study

**DOI:** 10.3390/s22052029

**Published:** 2022-03-05

**Authors:** Tino Prell, Manuela Uhlig, Steffen Derlien, Walter Maetzler, Hannah M. Zipprich

**Affiliations:** 1 Department of Geriatrics, Halle University Hospital, 06120 Halle, Germany; 2 Department of Neurology, Jena University Hospital, 07743 Jena, Germany; manuela.uhlig@uni-jena.de (M.U.); hannah.zipprich@med.uni-jena.de (H.M.Z.); 3 Institute for Physiotherapy, Jena University Hospital, 07743 Jena, Germany; steffen.derlien@med.uni-jena.de; 4 Department of Neurology, Kiel University, 24105 Kiel, Germany; w.maetzler@neurologie.uni-kiel.de

**Keywords:** fear of falling, Parkinson’s disease, mobile gait analysis, stride length, dual-task

## Abstract

Cognitive deficits and fear of falling (FOF) can both influence gait patterns in Parkinson’s disease (PD). While cognitive deficits contribute to gait changes under dual-task (DT) conditions, it is unclear if FOF also influences changes to gait while performing a cognitive task. Here, we aimed to explore the association between FOF and DT costs in PD, we additionally describe associations between FOF, cognition, and gait parameters under single-task and DT. In 40 PD patients, motor symptoms (MDS-revised version of the Unified Parkinson’s Disease Rating Scale, Hoehn and Yahr), FOF (Falls Efficacy Scale International), and Montreal Cognitive Assessment (MoCA) were assessed. Spatiotemporal gait parameters were recorded with a validated mobile gait analysis system with inertial measurement units at each foot while patients walked in a 50 m hallway at their preferred speed under single-task and DT conditions. Under single-task conditions, stride length (β = 0.798) and spatial variability (β = 0.202) were associated with FOF (adjusted R^2^ = 0.19, *p* < 0.001) while the MoCA was only weakly associated with temporal variability (adjusted R^2^ = 0.05, *p* < 0.001). Under DT conditions, speed, stride length, and cadence decreased, while spatial variability, temporal variability, and stride duration increased with the largest effect size for speed. DT costs of stride length (β = 0.42) and age (β = 0.58) explained 18% of the MoCA variance. However, FOF was not associated with the DT costs of gait parameters. Gait difficulties in PD may exacerbate when cognitive tasks are added during walking. However, FOF does not appear to have a relevant effect on dual-task costs of gait.

## 1. Introduction

Parkinson’s disease (PD) is characterized by a plethora of motor and non-motor symptoms that affect activities of daily living [1,2]. In particular, gait deficits are common in people with PD. PD gait is usually characterized by reduced step length, shuffling, delayed gait initiation, reduced speed, and—in later disease stages—by freezing of gait (FOG) [3,4,5]. Walking is a complex task and requires appropriate cognitive processes [6]. However, many people with PD develop cognitive deficits or PD dementia (PDD), including executive dysfunction, impaired processing speed, attentional, and language abilities [7]. A systematic review showed a direct association between the severity of cognitive impairment and gait deficits in people with dementia (i.e., walking speed decreased with progressing severity of dementia) [8], highlighting that the association between cognition and gait is not only relevant for people with PD. In line with this observation, gait deficits in PD exacerbate when cognitive tasks are added during walking (dual-task, DT) [9,10,11,12,13]. In particular, gait speed, stride length, gait asymmetry, and stride-to-stride variability are negatively influenced by DT in PD [12]. This negative effect of DT is often called DT cost, which is defined as the percentage change between single-task (ST) and DT gait parameters [14]. DT costs occur regularly, regardless of the mean level of ST gait speed and the type of DT [13,15].

Fear of falling (FOF) is a common and serious problem in patients with PD, which influences gait patterns [16,17]. FOF has been defined as ongoing concern about falling, low fall-related self-efficacy, and activity avoidance [18]. FOF restricts mobility, social participation, and quality of life [19,20]. FOF predicts future falls, and therefore, it is relevant to consider FOF for fall risk assessment in PD [21,22,23,24]. FOF in PD often arises from previous falls, near fall experiences, and disease progression [21,25]. In PD, FOF was reported to be associated with distinct motor function tests, turning metrics, impaired postural control, and fear of movement and activity resulting from a feeling of vulnerability to painful injury or re-injury [21,23,26,27], but also with gait speed, stride length, and functional balance performance [21,28,29,30]. Thus, while FOF affects normal gait (ST condition), it is less clear whether it can also modulate DT walking. Or, to paraphrase, it is currently unclear whether PD patients with high FOF have higher DT costs than those with low FOF. In a study of 24 de novo PD patients, DT gait speed was not influenced by FOF [31]. In contrast, in another study of PD patients with a mean disease duration of eight years, the variance of DT gait speed (walk and carry a tray) was partially explained by FOF; however, DT costs were not related to FOF [32].

In summary, in PD, normal walking (ST) is subject to numerous motor and non-motor influences (e.g., FOF), and cognitive impairments have been associated with DT walking deficits in PD. However, the extent to which DT walking is additionally influenced by FOF is poorly understood. With this study, we aimed to explore the association between FOF and DT costs in PD. We hypothesized that FOF increases DT costs independent from cognitive function. For this purpose, we used a mobile gait analysis system in order to provide reliable and objective data about gait characteristics [33,34]. These findings can help to develop tailored interventions for PD patients to prevent falls due to gait problems and cognitive deficits.

## 2. Materials and Methods

### 2.1. Study Design and Subjects

This cross-sectional study recruited PD patients from the ward of the Department of Neurology, Jena University Hospital, between January 2018 and July 2018. All patients gave written informed consent. The study was approved by local Ethics Committee (and has been performed in compliance with the Declaration of Helsinki.

Inclusion criteria were as follows: PD diagnosis according to Movement Disorder Society (MDS) diagnosis criteria, admission to hospital for PD multimodal complex treatment [35,36], able to walk 50 m without personal assistance, absence of severe dyskinesias affecting gait, adequate vision and/or hearing or successful use of corrective aids if required. Exclusion criteria were as follows: non-PD-related gait impairment, spasticity, cerebrovascular disorders, neuropathy, deep brain stimulation, levodopa/carbidopa enteral infusion, and apomorphine infusion. During the study period, 52 people with PD were admitted to the PD complex treatment in our hospital and screened for eligibility. Finally, 40 participants fulfilled inclusion criteria and were analyzed.

### 2.2. Assessments

All assessments were conducted during the medication ON phase at the beginning of the PD multimodal complex treatment. The following explanatory parameters were collected:

- Personal: age (metric, years), sex (nominal, male/female).
- Motor function: MDS-sponsored revision of the UPDRS III (MDS-UPDRS III, metric) [37], Hoehn and Yahr stage (multi-nominal, stage I to V), timed-up-and-go test (metric, sec), disease duration (metric, years), history of falls within the previous 6 months (nominal, yes or no), freezing of gait (nominal, present or absent), use of walking aid (nominal, yes or no).
- Cognition: Montreal cognitive assessment (MoCA, metric) [38]. Some patients had a MoCA score < 21 relating to PD dementia, however, this level of cognitive function was sufficient for their daily living (no legal guardian) [39].
- Mood: Beck’s depression inventory (BDI) II to measure degree of depressive symptoms (metric). FOF was measured using the Falls Efficacy Scale International (FES-I, metric) (α = 0.94) [40]. The FES-I is a self-report questionnaire, where the respondents answer how concerned they are about the possibility of falling in relation to 16 different activities (1 = not at all concerned to 4 = very concerned). The total FES-I ranges from 16 to 64, with higher values indicating more concerns about falling. FES-I total scores additionally were categorized into three groups: low (16–19 points), moderate (20–27), and high concerns about falling (28–64), according to previous works [41,42].

### 2.3. Gait Analysis and Test Protocol

Single-task: Participants were instructed to walk in preferred speed on a straight and flat 50 m-long hallway at the neurological inpatient clinic and were asked to turn at the respective end of the hallway without stopping. All participants were guarded by a research assistant, who walked behind the patient to prevent falls. Spatiotemporal gait parameters were automatically recorded by a validated mobile gait analysis system (RehaGait^®^, HASOMED GmbH, Magdeburg, Germany) [43,44]. RehaGait^®^ consists of two inertial sensors attached to the shoes; it streams raw data to a smart device application for real-time gait parameter calculation. A rule- and threshold-based pattern recognition algorithm was used to detect gait events (heel strike, full contact, heel off, toe off) [45], and a zero velocity assumption at full contact was used to minimize sensors integration drifts [46] For the analysis, the initial stride and all turning strides, including the stride before and after every turn, were excluded. The first 25 strides not excluded by the algorithm were used for this analysis.

DT: The assessments were repeated under DT condition (continue a combination of letters and numbers in chronological order and announce it aloud while walking, starting with A–1, B–2, C–3, etc.).

DT costs for gait parameters were calculated by: ((DT gait parameter–Single task gait parameter)/Single gait parameter) × 100 [47].

### 2.4. Statistical Analysis

The SPSS statistical computer package (version 25.0; IBM Corporation, Armonk, NY, USA) and JASP (version 0.16) were used for all statistical analyses. Prior to statistical analysis, data were checked for normality using the Shapiro–Wilk’s Test (*p* < 0.05). No outliers were removed. Descriptive analyses were used to describe clinical characteristics. Correlations were tested using Pearson’s correlation for normal distribution and Spearman’s correlation for non-normal distributed data. Groups were compared using one-way ANOVA or Kruskal–Wallis. To compare gait characteristics with and without DT, paired *t*-test or Wilcoxon test for paired samples were used. Finally, to determine factors associated with FES-I, MoCA or DT costs we used stepwise multiple linear regression or best subset regression (AIC).

Because also people with MCI or PDD were included, consistency and validity measures were calculated for the self-reported FES-I. Internal consistency of the FES-I was evaluated using the Cronbach’s coefficient α. Internal consistency was considered adequate if Cronbach’s coefficient α values were >0.70. Convergent validity was measured by calculating the Spearman correlation coefficient between FES-I and fall risk questionnaire. The Cronbach’s alpha of the FES-I in the normal cognition group (α = 0.945) was comparable to the MCI/PDD group (α = 0.944). The Spearman correlations between the FES-I and fall risk questionnaire did not differ between the group with normal cognition (r = 0.855, *p* = 0.001) and the MCI/PDD group (r = 0.734, *p* < 0.001) (Fisher’s z = 0.834, *p* = 0.40). Therefore, the self-report FES-I is valid and sound even in the presence of cognitive deficits.

Assuming a moderate effect of FOF on DT costs (d = 0.5), the sample size calculation revealed that 36 samples are necessary to achieve a significant result in one-sided *t*-test with power = 0.9 and alpha = 0.05.

The significance level for all analyses was set at *p* < 0.05.

## 3. Results

### 3.1. Descriptives

Detailed clinical characteristics of participants are given in Table 1.

According to the FES-I, 15.0% reported low concerns, 27.5% reported moderate concerns, and 57.5% reported great concerns about falling. People with higher FOF were more frequently in higher H&Y stages and had more often FOG (Supplement Table S1).

According to the MoCA, 27.5% had normal cognition, 30% had MCI, and 42.5% had PDD. Poorer cognitive function was associated with severe motor impairment as indicated by higher MDS-UPDRS III (Supplement Table S2). Comparing the MoCA subscores between people with normal (≥26 points) and impaired cognition (<26) revealed lower subscores in the MoCA subcategories “visuospatial/executive” (*p*  <  0.001; ε^2^  =  0.377), “language” (*p*  <  0.001; ε^2^  =  0.363), “abstraction” (*p*  =  0.022; ε^2^  =  0.141), and “memory” (*p*  <  0.001; ε^2^  =  0.299) in cognitively impaired people, whereas no difference was observed for categories “naming” (*p*  =  0.287; ε^2^  =  0.024), “attention” (*p*  =  0.116; ε^2^  =  0.066), and “orientation” (*p* = 0.234, ε^2^ = 0.0384) between groups (Kruskal−Wallis)

### 3.2. Gait Parameters Single-Task (ST)

We first describe how gait parameters were associated with the FES-I and MoCA under ST conditions. Gait parameters during normal walking/ST are given in Table 2. Stride length (*p* = 0.009), speed (*p* = 0.028), toe clearance (*p* = 0.05), and temporal variability (*p* = 0.001) differed between people with low, moderate and high FOF (Supplement Figure S1). In the univariate analyses, the FES-I correlated with stride length (r =−0.42, *p* = 0.006), speed (r =−0.43, *p* = 0.005), and toe clearance (r = −0.35, *p* = 0.029), but not with stride duration, cadence, spatial variability and temporal variability (Supplement Figure S2).

Multicollinearity was observed for stride length−speed and stride duration−cadence as indicated by a variance inflation factor above 10. Thus, only temporal variability, spatial variability, toe clearance, stride length, and stride duration were used as gait parameters in the following analyses. After entering these five gait parameters as independent variables into a linear regression on the FES-I (dependent variable), stride length (coefficient = −33.1, β = 0.798, *p* = 0.003) and spatial variability (coefficient = −0.72, β = 0.202, *p* = 0.11) were associated with FES-I (adjusted R^2^ = 0.19, *p* < 0.001) (stepwise forward selection, AIC).

In terms of cognitive function, no between-group differences were found for the gait parameters assessed. In the univariate analyses, the MoCA only correlated with temporal variability (r = 0.32, *p* = 0.047) (Figure 2). Temporal variability explained only 5% of the MoCA variance (*p* < 0.001). The MoCA subcategory “visuospatial/executive” correlated with stride length (r = 0.323, *p* = 0.048), spatial variability (r = 0.411, *p* = 0.010), and temporal variability (r = 0.375, *p* = 0.020); “abstraction” correlated with stride length (r = 0.342, *p* = 0.036) and speed (r = 0.359, *p* = 0.027), whereas “attention”, “language”, “memory”, “orientation” and “naming” did not correlate with gait parameters.

### 3.3. Gait Parameters under Dual-Task (DT)

**Figure 1:**
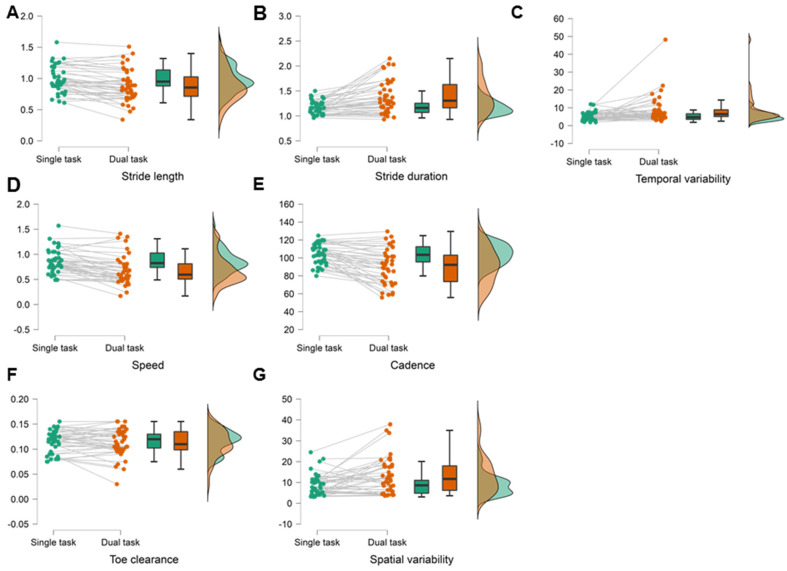
Changes of gait parameters between single- and dual-task condition. (**A**) stride length decreased (*p* < 0.001), (**B**) stride duration (*p* < 0.001) increased, (**C**) temporal variability (*p* < 0.001) increased, (**D**) speed (*p* < 0.001) decreased, (**E**) cadence decreased (*p* < 0.001), (**F**) toe clearance (*p* = 0.405) remained unchanged, (**G**) spatial variability (*p* = 0.003) increased under dual task with highest effect size for speed.

In a second step, we describe how gait parameters change under DT conditions. During the DT condition, participants walked slower with shorter steps. Speed, stride length, and cadence decreased, while spatial variability, temporal variability, stride duration increased under DT with the largest effect size for speed. Toe clearance remained unchanged during DT (Table 2, Figure 1). DT costs of each gait parameter are given in Table 2.

### 3.4. Association between FES-I and DT Costs

**Figure 2:**
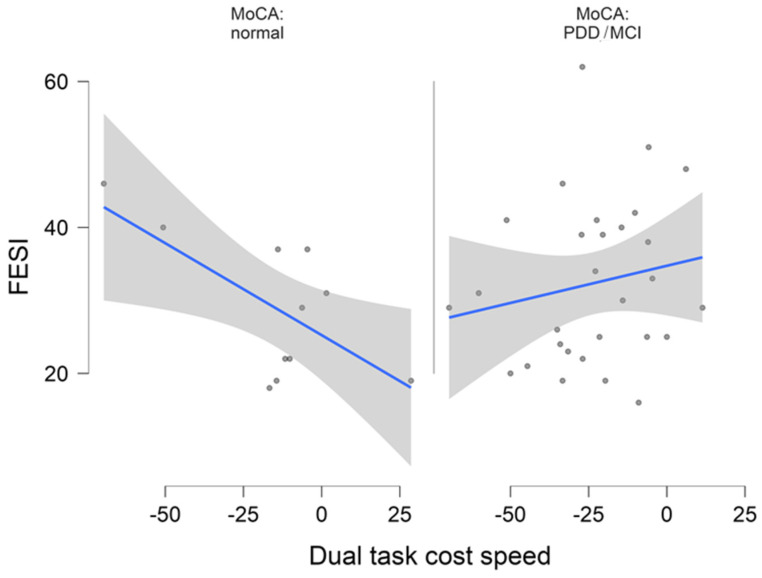
Flexplot.

Finally, we explored the association between DT costs, FES-I, and the MoCA. DT costs of gait parameters did not significantly differ between people with low, middle, and high FOF according to the FES-I (Kruskal–Wallis, *p* > 0.05), and DT costs of gait parameters did not significantly correlate with the FES-I. Accordingly, DT costs of gait parameters explained none of the FES-I variance based on the given *R*^2^ value. As illustrated in Figure 2, the relationship between FES-I and DT costs differed depending on cognitive state. In people with normal cognitive function (MoCA > 26) the FES-I correlated not significantly (r = −0.265, *p* = 0.43) with DT cost of speed and in people with MCI or PDD the FES-I correlated non significantly only weakly positive (r = 0.193, *p* = 0.315) with DT cost of speed. However, these findings have to be interpreted cautiously, due to the lower sample size in the normal cognition group.

The relationship between FES-I and DT costs for speed differed depending on cognitive state. In people with a MoCA > 26 the FES-I correlated not significantly negatively (r = −0.265, *p* = 0.43) with DT cost of speed. In people with poorer cognitive function (mild cognitive impairment, MCI or Parkinson’s disease dementia, PDD) the FES-I correlated non-significantly only weakly positive (r = 0.193, *p* = 0.315) with DT cost of speed.

Of note, there were no significant group effects between MoCA groups (normal, MCI, PDD) in DT costs of the gait parameters (Kruskal–Wallis, *p* > 0.05). There was a weak correlation between DT cost of stride length and MoCA (r = 0.317, *p* = 0.046). In the linear regression, DT costs of stride length (coefficient = 0.077, *p* = 0.046) explained 8% of the MoCA variance (*p* < 0.001, best subset regression, AIC). After correction for age, disease duration and BDI, the DT costs of stride length (β = 0.42, *p* = 0.047) and age (β = 0.58. *p* = 0.02) remained associated with the MoCA (*p* < 0.01, corrected R^2^ = 0.18, best subset regression, AIC).

## 4. Discussion

In this study, we compared gait spatiotemporal and variability measures, cognitive, and FOF measures in terms of ST and DT walking conditions in people with PD.

The majority of our participants reported various degrees of FOF and had cognitive deficits according to the MoCA. This is not surprising because non-motor symptoms, such as depression, anxiety, and cognitive decline, are common in PD [5]. We first analyzed gait parameters under normal walking/ST conditions in order to better situate our results within the existing literature. During ST walking, we found that FOF-related gait was mainly associated with shorter stride length (and correspondingly decreased speed). This agrees with the study of 79 PD patients by Bryant et al., who found that gait speed and stride length for forward-walking were lower for those with a high level of FOF compared to those with a low level of FOF [21]. In another study, FOF remained significantly associated with decreased walking speed also after correction for sex, age, motor impairment (UPDRS III), and depression (HADS-D) [32]. Gait changes in people with FOF may be due to actual functional PD-related deficits, or FOF itself could modify gait. Both mechanisms are likely to occur simultaneously and mutually reinforcing [21,48]. As in earlier studies, we did not find a significant association between previous falls and FOF, highlighting that FOF could appear as a fall-independent condition [18,30,42].

Examining the impact of cognition on gait during simple walking (ST) is challenged by age-related gait changes that also occur in healthy older adults. It seems that different cognitive domains (e.g., executive function, attention) differentially influence temporal and postural aspects of gait [49,50,51,52,53]. For PD, little is known about the relationship between ST walking and cognitive function. In a study of 45 people with mild to moderate PD, for the single-task condition, stride length and gait speed were associated with processing speed measures, and step width variability was associated with executive function and attention measures [51]. We did not observe a strong effect of overall cognitive performance (MoCA) on the assessed gait parameters. A similar result was found in another sensor-based gait analysis where cognitively impaired PD patients did not have significantly different stride length and gait speed compared with PD patients without cognitive impairment [14]. In line with the study by Stegemöller et al., in our cohort, the MoCA subcategory “visuospatial/executive” correlated with spatial gait and variability parameters [51]. Attention did not significantly correlate with gait parameters. However, the methods in our study and in the study by Stegemöller et al. differed in terms of the cognitive test used, gait analyses, and cohort characteristics.

The association between cognition and gait is frequently studied under DT conditions, i.e., gait changes while completing a cognitive task [32,54,55,56,57]. DT can reveal a lack of automaticity and increased cognitive demands during walking [32]. Furthermore, among healthy adults, the DT walking deficits increase with age; however, in PD, there is consistently a greater DT walking deficit than in healthy, age-matched individuals [58]. DT gait speed has been associated with several motor factors (Hoehn and Yahr stage, UPDRS III, freezing of gait) and cognitive factors (executive function, set-shifting, and attention) [58]. In line with our findings, DT was accompanied by reduced gait speed and stride length and increased variability [10,53,55,59,60,61]. It was hypothesized that decreasing speed or stride length is a protective response to DT in both PD and healthy older adults [57].

Thus, it is evident that gait speed decreases with increasing cognitive impairment and that this effect can be measured by DT. However, the question was open whether the extent of speed reduction under DT (DT speed cost) allows conclusions to be drawn about the severity of the cognitive impairment. In order to answer this question, Gaßner et al. investigated cognitive function, single-task, and DT gait performance in 67 PD patients [14]. They hypothesized that PD patients with cognitive deficits show higher DT costs in gait parameters. However, they did not reveal correlations between DT costs of gait parameters and cognitive performance assessed with MoCA. In their study, DT costs of distinct gait parameters (stride length, swing time variability, and toe clearance) explained only 8% of the cognitive variance, suggesting that DT gait performance is not relevantly indicative for cognitive impairment in PD [14]. This is in line with our findings. We also found that DT costs of stride length explained 8% of the MoCA variance. Together with age, the DT costs of stride length explained 18% of the MoCA variance. Therefore, we were able to replicate the findings by Gaßner et al.

Finally, we aimed to answer how FOF and DT costs are related to each other in PD. We could not confirm our hypothesis that FOF increases DT costs. Although FOF was clearly associated with gait parameters during ST walking in our and other abovementioned studies, DT costs of gait parameters were not significantly related to FOF in the entire cohort. As there was neither a significant correlation between FOF and MoCA nor between FOF and gait DT costs, we assume that FOF does also not have a mediating/indirect effect on gait DT costs. This suggests that FOF has no remarkable influence on changes of gait while performing a cognitive task.

What can be concluded for clinical practice? Falls are common in people who are cognitively impaired. In particular, for PD, disentangling the mechanism and contribution of cognitive problems to falls and fall risk may open new treatment approaches [62]. Several PD clinical rehabilitation programs and therapies consider DT interventions as promising tools to reduce falls [63,64] because the use of DT during training seems to have benefits related to gait and balance parameters [65]. However, more studies are necessary to determine in which PD patient group the DT training has benefited [64]. For designing training programs, it is, however, necessary to know relevant cofactors that might influence DT costs. Our study indicates that the highly prevalent FOF in people with PD does not contribute to DT costs. Therefore, one can assume that FOF does not increase fall risk by aggravating DT costs in cognitively impaired PD patients. Rather FOF is to be regarded as an independent issue that might aggravate gait problems in PD.

Our study faces limitations. First, we focused on straight walking on a flat corridor. It may thus be promising to evaluate gait in more complex settings and movement behaviors such as turning and transfers. Second, this study focused on gait parameters that are relevant for current rehabilitation approaches for PD. There are more potentially independent gait parameters extractable with such inertial measurement unit-based technique, and it is possible that a more refined analysis approach could unveil additional associations between specific gait parameters and FOF. We do acknowledge that there may still be other influential factors for FOF that deserve consideration, such as level of physical activity and physical environmental barriers. Another limitation is that, due to the cross-sectional design, we cannot make causal statements.

## 5. Conclusions

FOF does not seem to modulate gait DT cost to a relevant extent. Moreover, our data suggest that FOF does not exacerbate DT effects in cognitively impaired people with PD.

## Figures and Tables

**Table 1:** Demographical and clinical characteristics.

	Median	Mean	SD	IQR	*p* (Shapiro–Wilk)
Age (years)	72.50	70.65	8.83	14.50	0.015
Disease duration (years)	9.00	8.88	4.90	8.25	0.132
MDS-UPDRS III (0–132)	26.00	29.92	14.05	19.50	<0.001
Montreal Cognitive Assessment (MoCA) (0–30)	22.00	22.08	3.94	7.00	0.229
Beck’s depression inventory II (BDI) (0–63)	12.00	12.92	7.22	6.75	0.018
Timed-up-go-test (s)	12.70	16.99	12.48	10.41	<0.001
Falls Efficacy Scale International (16–64)	29.50	31.45	10.78	17.25	0.041
		**n**	**%**		
Sex	female	17	42.5		
	male	23	57.5		
Hoehn and Yahr stage	1	6	15.0		
	2	6	15.0		
	3	20	50.0		
	4	8	20.0		
Presence of freezing of gait (FOG)	no FOG	27	67.5		
	FOG	13	32.5		
Use of walking aid	no	29	72.5		
	yes	11	27.5		
Fall(s) within last 6 months	No	19	47.5		
	Yes	21	52.5		

**Table 2:** Descriptive Statistics for gait measures (single task, dual task) and dual-task cost.

	Single Task		Dual Task		Paired Test (Single Task–Dual Task)		Dual-Task Cost	
	Mean	SD	Mean	SD	*p*	Effect Size	Mean	SD
Stride duration (s)	1.17	0.13	1.39	0.33	<0.001 ^#^	−0.804	−21.30	21.27
					<0.001 ^§^	−0.896		
Stride length (m)	0.99	0.22	0.89	0.26	<0.001 ^#^	0.720	18.73	23.92
					<0.001 ^§^	0.693		
Speed (m/s)	0.86	0.24	0.69	0.29	<0.001 ^#^	0.941	−10.68	15.35
					<0.001 ^§^	0.872		
Cadence (steps/min)	103.95	11.46	90.77	19.84	<0.001 ^#^	0.891	−13.00	14.16
					<0.001 ^§^	0.868		
Toe clearance (m)	0.12	0.02	0.11	0.03	0.205 ^#^	0.204	−3.11	18.36
					0.405 ^§^	0.161		
Variability spatial (%)	9.01	5.14	13.42	8.76	0.001 ^#^	−0.542	110.62	218.39
					0.003 ^§^	−0.534		
Variability temporal (%)	5.13	2.39	8.83	7.90	0.006 ^#^	−0.460	81.43	155.02
					<0.001 ^§^	−0.585		

Paired test: ^#^ Student’s *t* Test, ^§^ Wilcoxon test.

## Data Availability

The data used to support the findings of this study are available from the corresponding author upon request for scientific purposes only.

## Supplementary Materials

The following supporting information can be downloaded at: https://www.mdpi.com/article/10.3390/s22052029/s1, Table S1. Cross table for different degrees of fear of falling according to the Falls Efficacy Scale International (FES-I); Table S2. Cross table for different cognitive states according to the Montreal cognitive assessments (MoCA); Figure S1. Boxplots for group comparisons; Figure S2. Univariate correlation plots.
