# Supplementary material for: Fear of Falling Does Not Influence Dual-Task Gait Costs in People with Parkinson’s Disease: A Cross-Sectional Study
Source: Sensors (Basel). 2022 Mar 5;22(5):2029. doi: 10.3390/s22052029 (PMC8914753; doi:10.3390/s22052029)
Supplement: Supplementary file 1 [file sensors-22-02029-s001.zip › sensors-1593326-supplementary.pdf]

# Supplement

# Fear of Falling Does Not Influence Dual-Task Gait Costs in People with Parkinson's Disease: A Cross-Sectional Study

**Tino Prell [1,*], Manuela Uhlig [2], Steffen Derlien [3], Walter Maetzler [4] and Hannah M. Zipprich [2]**

1 Department of Geriatrics, Halle University Hospital, 06120 Halle, Germany
2 Department of Neurology, Jena University Hospital, 07743 Jena, Germany; manuela.uhlig@uni-jena.de (M.U.); hannah.zipprich@med.uni-jena.de (H.M.Z.)
3 Institute for Physiotherapy, Jena University Hospital, 07743 Jena, Germany; steffen.derlien@med.uni-jena.de
4 Department of Neurology, Kiel University, 24105 Kiel, Germany; w.maetzler@neurologie.uni-kiel.de
* Correspondence: Tino.prell@uk-halle.de

**Table S1.** Cross table for different degrees of fear of falling according to the Falls Efficacy Scale International (FES-I).

| | Low Concerns | Moderate Concerns | High Concerns | Test Statistics |
|---|---|---|---|---|
| | (N = 6) | (N = 11) | (N = 23) | |
| Age (years) | 53.8 60.0 75.1 | 67.3 76.0 80.5 | 64.3 71.0 78.0 | $F_{1,38} = 1.17$, $P = 0.29$ |
| Disease duration (years) | 0.9 8.0 14.2 | 3.3 8.0 12.5 | 5.2 10.0 13.8 | $F_{1,38} = 1.03$, $P = 0.32$ |
| MDS-UPDRS III (0–132) | 17.8 24.5 41.2 | 15.7 24.0 29.5 | 21.2 28.0 40.0 | $F_{1,38} = 1.35$, $P = 0.25$ |
| Montreal Cognitive Assessment (MoCa) (0–30) | 18.8 25.5 27.1 | 19.3 22.0 24.7 | 19.0 22.0 25.7 | $F_{1,38} = 0.93$, $P = 0.34$ |
| Beck´s depression inventory II (BDI) (0–63) | 7.8 11.0 14.2 | 5.5 9.0 15.0 | 10.0 13.0 16.7 | $F_{1,38} = 2.05$, $P = 0.16$ |
| Timed-up-go-test (s) | 5.9 8.9 9.1 | 8.8 12.7 15.5 | 10.4 16.7 27.6 | $F_{1,37} = 10.20$, $P < 0.01$ |
| Falls Efficacy Scale International (16–64) | 17.8 19.0 19.0 | 22.0 23.0 25.0 | 31.3 39.0 41.8 | $F_{1,38} = 141.48$, $P < 0.01$ |
| Gender: male | 0.7 4/6 | 0.6 7/11 | 0.5 12/23 | $\chi = 0.64$, $P = 0.73$ |
| FOG: FOG | 0.0 0/6 | 0.2 2/11 | 0.5 11/23 | $\chi = 6.38$, $P = 0.04$ |
| Walking aids: yes | 0.0 0/6 | 0.2 2/11 | 0.4 9/23 | $\chi = 4.32$, $P = 0.12$ |
| Falls: Yes | 0.3 2/6 | 0.5 5/11 | 0.6 14/23 | $\chi = 1.75$, $P = 0.42$ |
| Hoehn & Yahr stage | | | | $\chi = 10.37$, $P = 0.11$ |
| 1 | 0.5 3/6 | 0.2 2/11 | 0.0 1/23 | |
| 2 | 0.2 1/6 | 0.2 2/11 | 0.1 3/23 | |
| 3 | 0.3 2/6 | 0.5 6/11 | 0.5 12/23 | |
| 4 | 0.0 0/6 | 0.1 1/11 | 0.3 7/23 | |

**Table S2.** Cross table for different cognitive states according to the Montreal cognitive assessments (MoCA).

| | **Normal** | **MCI** | **PDD** | **Test Statistic** |
|---|---|---|---|---|
| | (N = 11) | (N = 12) | (N=17) | |
| Age (years) | 60.0 64.0 73.3 | 69.4 72.5 78.6 | 65.3 76.0 78.0 | $F_{1,38} = 2.49$, $P = 0.12$ |
| Disease duration (years) | 3.0 8.0 12.7 | 5.4 9.0 13.6 | 5.0 10.0 14.0 | $F_{1,38} = 0.41$, $P = 0.53$ |
| MDS-UPDRS III (0–132) | 20.0 22.0 25.7 | 15.4 26.5 40.2 | 22.3 29.0 43.3 | $F_{1,38} = 3.55$, $P = 0.07$ |
| Montreal Cognitive Assessment (MoCa) (0–30) | 26.0 27.0 27.8 | 22.0 23.0 23.6 | 18.0 19.0 19.3 | $F_{1,38} = 292.53$, $P < 0.01$ |
| Beck´s depression inventory II (BDI) (0–63) | 8.0 11.0 14.0 | 6.2 9.5 13.6 | 9.7 13.0 16.3 | $F_{1,38} = 1.40$, $P = 0.24$ |
| Timed-up-go-test (s) | 6.5 9.7 18.4 | 8.8 13.4 18.0 | 9.7 13.5 23.2 | $F_{1,37} = 1.88$, $P = 0.18$ |
| Falls Efficacy Scale International (16–64) | 19.5 29.0 37.0 | 24.4 27.5 43.1 | 21.7 33.0 40.3 | $F_{1,38} = 0.59$, $P = 0.45$ |
| Gender: male | 0.4 4/11 | 0.6 7/12 | 0.7 12/17 | $\chi = 3.21$, $P = 0.20$ |
| FOG: FOG | 0.3 3/11 | 0.5 6/12 | 0.2 4/17 | $\chi = 2.44$, $P = 0.30$ |
| Walking aids: yes | 0.1 1/11 | 0.3 4/12 | 0.4 6/17 | $\chi = 2.59$, $P = 0.27$ |
| Falls: Yes | 0.5 6/11 | 0.6 7/12 | 0.5 8/17 | $\chi = 0.38$, $P = 0.83$ |
| Hoehn & Yahr stage | | | | $\chi = 12.30$, $P = 0.06$ |
| 1 | 0.5 5/11 | 0.0 0/12 | 0.1 1/17 | |
| 2 | 0.1 1/11 | 0.2 3/12 | 0.1 2/17 | |
| 3 | 0.4 4/11 | 0.5 6/12 | 0.6 10/17 | |
| 4 | 0.1 1/11 | 0.2 3/12 | 0.2 4/17 | |

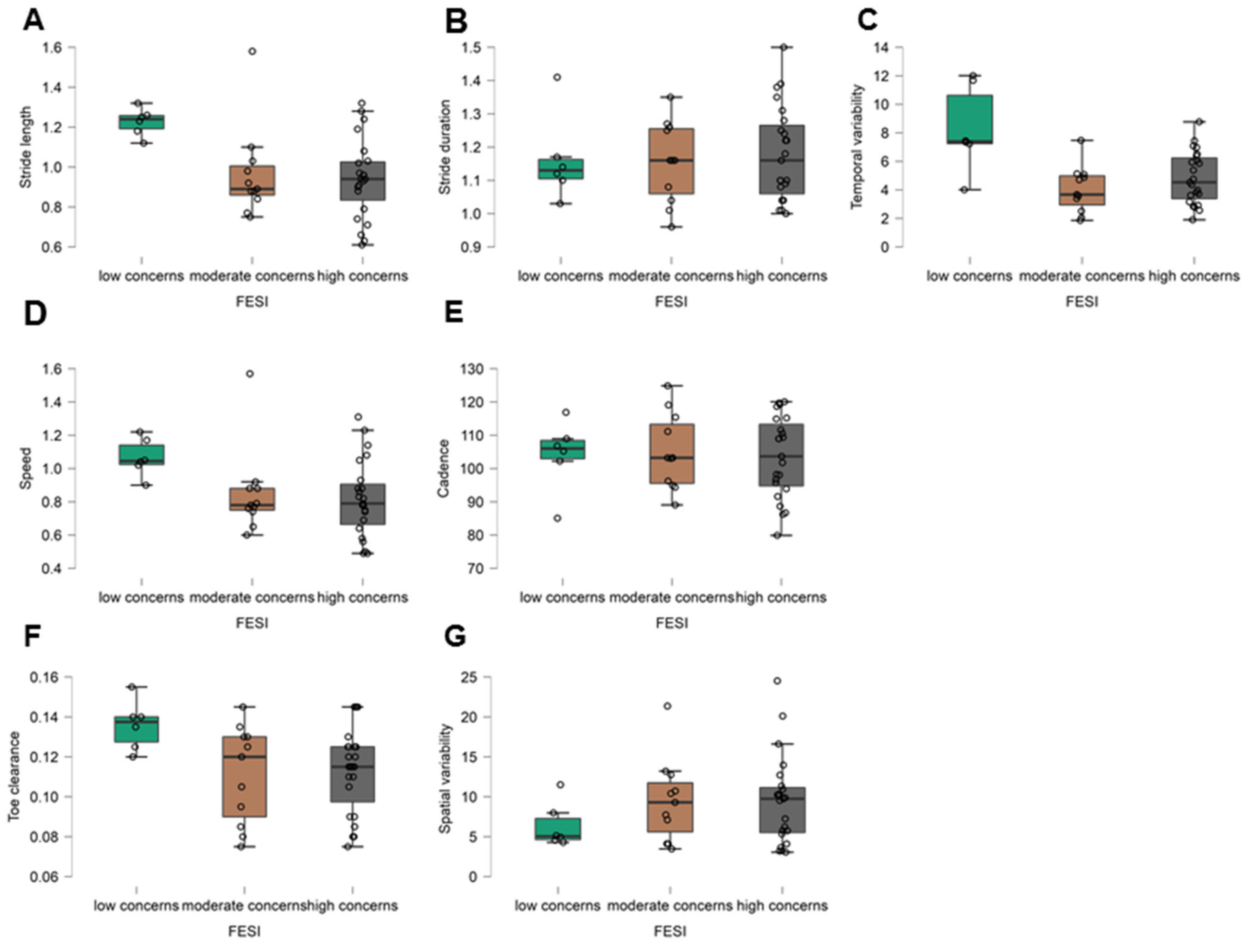


**Figure S1.** Boxplots for group comparisons. Between group differences among people with low, moderate and high fear of falling according to the Falls Efficacy Scale International (FES-I) displayed for **A**) stride length ($p = 0.009$), **B**) stride duration, **C**) temporal variability ($p = 0.001$), **D**) speed ($p = 0.028$), **E**) cadence, **F**) toe clearance ($p = 0.05$), and **G**) spatial variability. .

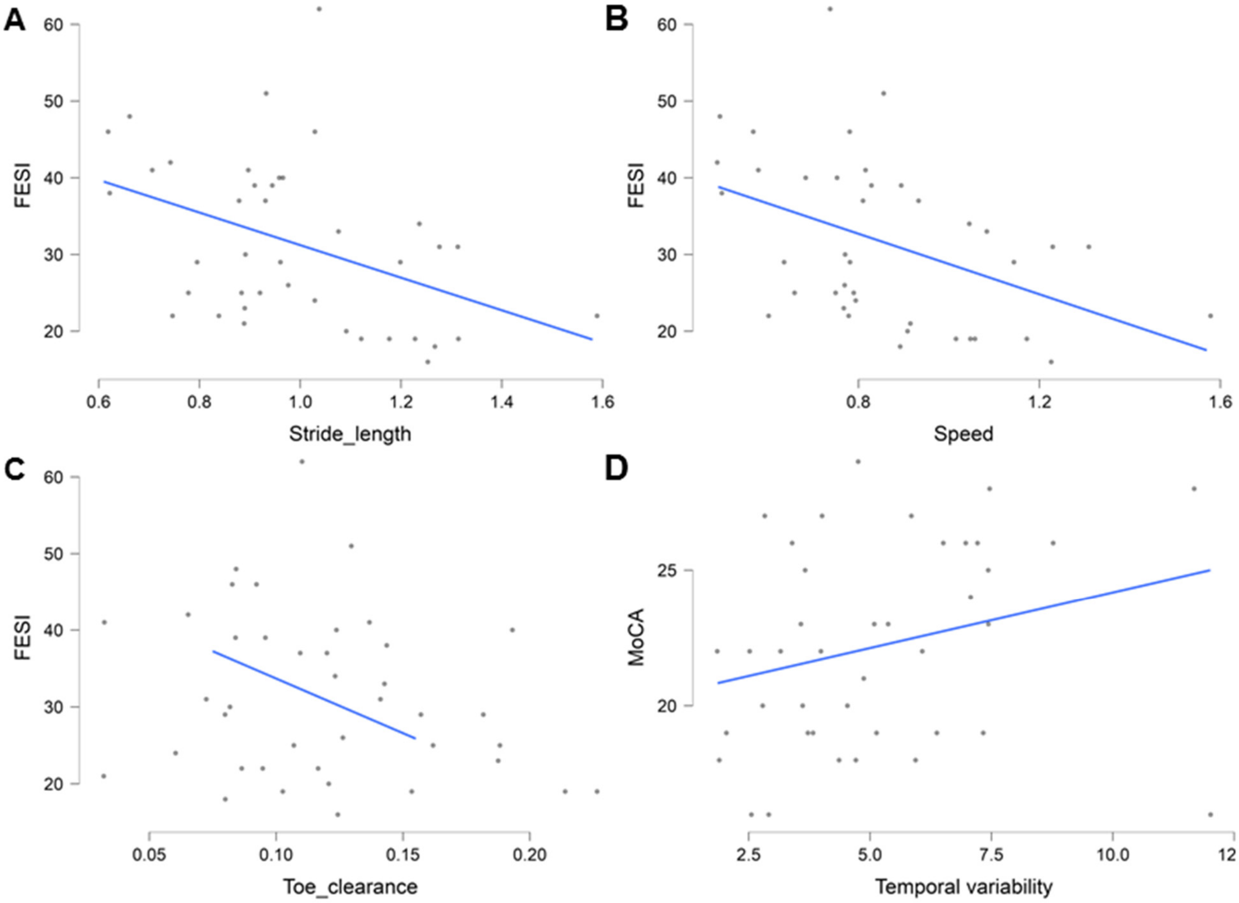


**Figure S2.** Univariate correlation plots. In the univariate analyses the Falls Efficacy Scale International (FES-I) correlated significantly with **A**) stride length (r = -0.42, $p$ = 0.006), **B**) speed (r = -0.43, $p$ = 0.005), and **C**) toe clearance (r=-0.35, $p$ = 0.029). **D**) The Montreal cognitive assessment correlated significantly only with temporal variability (r = 0.32, $p$ = 0.047).
